# Reactive molecular dynamic simulations on the gas separation performance of porous graphene membrane

**DOI:** 10.1038/s41598-017-14297-w

**Published:** 2017-11-29

**Authors:** Somaye Esfandiarpoor, Mostafa Fazli, Masoud Darvish Ganji

**Affiliations:** 1 Department of Applied Chemistry, Semnan University, Semnan, Iran; 2 Department of Nanochemistry, Faculty of Pharmaceutical Chemistry, Pharmaceutical Sciences Branch, Islamic Azad University, Tehran, Iran; 3 Department of Chemistry, Qaemshahr Branch, Islamic Azad University, Qaemshahr, Iran

## Abstract

The separation of gases molecules with similar diameter and shape is an important area of research. For example, the major challenge to set up sweeping carbon dioxide capture and storage (CCS) in power plants is the energy requisite to separate the CO_2_ from flue gas. Porous graphene has been proposed as superior material for highly selective membranes for gas separation. Here we design some models of porous graphene with different sizes and shape as well as employ double layers porous graphene for efficient CO_2_/H_2_ separation. The selectivity and permeability of gas molecules through various nanopores were investigated by using the reactive molecular dynamics simulation which considers the bond forming/breaking mechanism for all atoms. Furthermore, it uses a geometry-dependent charge calculation scheme that accounts appropriately for polarization effect which can play an important role in interacting systems. It was found that H**-**modified porous graphene membrane with pore diameter (short side) of about 3.75 Å has excellent selectivity for CO_2_/H_2_ separation. The mechanism of gas penetration through the sub-nanometer pore was presented for the first time. The accuracy of MD simulation results validated by valuable DFT method. The present findings show that reactive MD simulation can propose an economical means of separating gases mixture.

## Introduction

Due to its abundance, easy synthesis, and nonpolluting properties, hydrogen has been considered to be the most promising energy source, nowadays. Hydrogen energy technologies can play a special role in solving problems related to the environment sectors^1–5^. Because of increasing demand for purified hydrogen, extensive research studies have been devoted to the advancement of the technologies for generation and purification of hydrogen with higher efficiency and lower production cost^6^.

The universal carbon dioxide emissions, on the other hand, are expected to increase as long as fossil fuels continue to be the main energy source for mankind. Numerous industrial processes emit CO_2_ streams with different compositions, which require corresponding CO_2_ separation processes. CO_2_ a main greenhouse gas (GHG), is discharged into the atmosphere via different processes: namely the fossil fuel combustion (petroleum, coal, and natural gas)^7^. Accounting for about 60% of the heat trapped in the atmosphere, CO_2_, potentially, has the greatest inclusion in climate change, among all GHGs. The increase in air temperature along with changes in precipitation patterns, has led to an increase in the number and strength of natural disasters; like floods, droughts, hurricanes, extensive melting of ice and snow, and an increase in medium sea levels^8^. These affect directly and indirectly on human life and ecological systems on the planet earth. Increasing the general consciousness on the subject has urged researchers and policy makers, to study climate change and find a solution to lessen this threat.

There are a number of ways to alleviate the GHGs emission to the atmosphere that among them replacing common fossil fuel based energies with more environmentally compassionate sources (renewable energy), and the CO_2_ capture and storage (CCS) have the most importance. In spite of researches about renewable energy, the fossil fuels are the main energy for mankind and remains as predicted, the main energy for at least the next two decades. So, CCS is the best way to lessen climate change^9^. CO_2_ capture is currently a topical issue in environmental protection and sustainable development^10^ and has been intensively researched and developed to survive with climate change, by reducing atmospheric CO_2_ concentration^11,12^. CO_2_ can be obtained from numerous sources including fossil fuel power plants, refineries, oil and gas production sites, iron and steel factories, cement and other chemical plants. CO_2_ capture can be classified into four main techniques: 1- pre-combustion 2- post-combustion 3- oxy-fuel and 4-electrochemical separation^13–16^. Pre-combustion capture uses new gasification method to create combustible gas and then capture the CO_2_ before burning for power. In pre-combustion capture, the CO_2_ concentration is high, which lead to a higher driving force for the CO_2_ separation and potential for cost savings in the absence of compression necessity. For pre-combustion capture, the carbonaceous fuel is first converted to a form amenable to CO_2_ capture.

The fuel gas called synthetic gas (syngas) is composed of mainly H_2_ and CO_2_ with a trace amount of H_2_O and H_2_S gases. The high concentration of CO_2_ (20–40%) in the fuel gases needs to be eliminated before the combustion. The syngas gas is a mixture of CO, H_2_, H_2_O, CO_2_, and other components. There are some impurities in the syngas, which need to be eliminated and then through a two-step water gas shift, carbon monoxide would convert to carbon dioxide^16,17^. The produced mixture is mainly composed of CO_2_ and H_2_. Then the mixture goes through a CO_2_ capture system, to create a nearly-pure hydrogen stream. This hydrogen flow goes into a combined cycle power plant to generate electricity.

There are different separation techniques in detail, for CO_2_ capture: a) adsorption, b) absorption, c) cryogenic distillation, d) membranes, e) gas hydrates, and f) chemical looping^18^. Except for cryogenic separation, the rest of these methods require some material as carriers. Currently, absorption, via solvent scrubbing, is a deep-rooted CO_2_ separation approach, which has been implemented broadly in chemical and petroleum industries. Cryogenic distillation is based on the principales of separation based on cooling and condensation. It has been used in liquid separations for a long time. This technique is hypothetically very useful for CO_2_ separation; but the substantial energy requirement makes it less desirable for most applications. Membranes separate gases based on the contrasts in physical and chemical interactions between different gases and the membrane materials. This would allow some components to pass preferentially through the membranes based on size (kinetic) and affinity (thermodynamics)^19^. Membrane technology can be a powerful tool for CO_2_ capture process by decreasing equipment size and lowering energy requirement.

Generally, for industrial gas separation there are three major processes: pressure swing adsorption (PSA), cryogenic distillation and membrane gas separation^8^. Traditional systems for gas separation in industry consume a very high energy cost^4^ and lead to environmental problems. The use of membrane system^20^ as an efficient separation for the gas mixtures without any phase change clearly lessens the energy cost compared to traditional systems. The main advantages that membrane technology presents over other gas separation methods like pressure swing adsorption and cryogenic distillation are its low power usage and costs, simplicity in operation and its compactness and portability^1^. The good separation membrane must have controllable pore size^21^, stable structure, and efficient permeability. Numerous studies have been devoted to evaluate potential applications of graphene for suitable gas separation^22–26^. Recent theoretical work by Jiang *et al*. has proposed porous graphene^27^ as a promising material for highly selective membranes. Graphene is an extended honeycomb network of *sp* ^2^-hybridized carbon atoms and the first example of a close-packed two-dimensional (2D) crystalline material isolated in nature and has emerged to become an exciting new nanomaterial of carbon with many novel properties^28,29^. So, graphene, the single-atom-thick planar membrane^28,29^ and the strongest structure^30^ that exists so far in the earth, becomes interesting for its potential applications to be a good membrane. The use of graphene as gas storage has also been an active area of research, due to their extended π-conjugation and high surface area^31,32^.

The gas separation performance of porous graphene membrane can be investigated by creating the sizes and shapes of their pores and by the chemical functionalization of graphene. For example, Liue *et al*.^33^ found that gas mixtures can effectively been separated with porous graphene via the size exclusion using sub-nanometer pore rims. Also, the chemical functionalization of graphene pore rims is effective on gas permeability and selectivity. Xue *et al*.^34^ demonstrated that porous graphene membrane with N-passive pores has a strong electrostatic force for CO_2_ compared with N_2_ when permeating through the pores. In 2016 Wang *et al*. used both molecular dynamic simulations and DFT calculations to investigate the gas separation performance of H-passive nanoporous graphene membrane. They considered a mixture of CO_2_/N_2_ gas molecules and three different pore sizes for the H-passive membrane. They found that H-pore-13 (among H-pore-10 and H-pore-16) has a higher interaction energy with CO_2_ in compared with N_2_. Also the barrier energy of CO_2_ (0.19 eV) is much more than that of N_2_ (0.05 eV) so N_2_ can permeate through H-pore-13, whereas CO_2_ cannot^35^.

The current study entails the permeability and selectivity of pore graphene with different functional atoms (C and H) and different pore size for CO_2_/H_2_ gas mixture. We employ reactive molecular dynamics simulation (a realistic aspect of our simulation) and DFT calculations in order to gain insights into the penetration and interaction properties of passing molecules through the pore. Our results demonstrate that permeability and selectivity of considered pores are highly sensitive to the functional group atoms as well as the pore size. It was found that the H-passive pore graphene membrane with diameter of **3.75** Å exhibits a high selectivity for CO_2_/H_2_ separation. The mechanism of penetrated molecules across the considered pore has also been investigated and validated by using the dispersion corrected DFT calculations. It is expected that this study could provide useful information for CO_2_ gas capture/separation from H_2_ as well as environment protection.

## Computational Procedures

The performance of an idealized graphene membrane was evaluated for H_2_/CO_2_ gas separation using both quantum mechanical calculations and molecular dynamics (MD) simulations. DFT calculations were employed to optimize the structure of graphene and explore the energy barriers and potential energy surface of H_2_/CO_2_ molecules passing through sub-nanometer pores created in a graphene sheet. DFT calculations were performed using the *ab initio* package SIESTA, which is based on the localized basis set and the method of pseudo potentials^33^. In this software the electronic wave functions are constructed by linear combination of locally-confined atomic orbitals (LCAO’s)^36^. Norm-conserving pseudo potentials of Troullier–Martins^37^ with the valence electron configurations of all considered atoms were implemented. Dispersion interactions should be important for the systems under study and therefore we utilize the DFT-D2 method in order to consider the respective interactions. The DFT-D approach has been extensively tested on numerous systems^38^ including the physisorption of small molecules to graphene sheets^39,40^ and the adsorption of H_2_ within metal**-**organic framework materials^41^. The optimization procedure has been carried out with SCF tolerance of 10^−5^ eV and residue force on each atom was set to 0.02 eV/Å. The Monkhorst-Pack approach was used to represent the Brillouin zone by *k*-points of 5 × 5 × 1 meshes for the graphene sheets. We utilized a vacuum thickness of 25 Å along the z direction of the graphene layers. All the atomic coordinates including hydrogen and carbon atoms of the pore membranes were fully optimized.

The values for the interaction energies, *E*_int_, and the corresponding barrier energies, *E*_barr_ are calculated from following equations:

1 $${E}_{{\rm{int}}}={E}_{{\rm{M}}/\mathrm{Graph}}-{E}_{{\rm{Graph}}}-{E}_{{\rm{M}}}$$

2 $${E}_{{\rm{barr}}}={E}_{{\rm{int}}{\rm{.max}}}-{E}_{{\rm{int}}{\rm{.min}}}$$

where *E*_Graph_ and *E*_M_ correspond to total energies of the relaxed graphene and isolated H_2_/CO_2_ molecule, respectively. *E*_M/Graph_ is the total energy of the optimized complex. *E*_int.max_ and *E*_int.min_ are defined as maximum and minimum values of the interaction energies.

In some cases, to verify geometry parameters as well as adsorption properties of interacting entities the hybrid density functional B3LYP^42,43^ calculations were performed by using the modern electronic structure package, ORCA^44^. We used the def2-TZVP (split-valence triple-zeta) basis set^45^ for all atoms. The Grimme approach using atom pair-wise additive schemes^46^, so-called DFT-D3 method, was utilized to consider the dispersion corrections for the long range non-bonding van der Waals (vdW) interactions. The DFT-D3 method has been shown to be well performed with Becke-type exchange such as BLYP or B3LYP functionals^47^.

Molecular mechanics (MM) calculations with model potentials were used to calculate gas passing barriers and compared with the DFT calculations to assess the accuracy of the classical potentials. All MM calculations were carried out with General Utility Lattice Program (GULP) 4.0 to simulate separation of gas mixtures^40^. GULP supports geometry optimization and MD simulations of molecules, clusters, and 2D-/3D-systems using a wide range of potential models that span both the inorganic and organic fields. The potentials include shell model, embedded atoms (for metals), and bond order/reactive force fields. The range of potential models means that structures as diverse as zeolites and metallic films on semiconductor substrates can be modeled.

We used a superior reactive force field so-called as ReaxFF. At the center of the ReaxFF potential lies a bond order/bond energy relationship. Bond orders were obtained from interatomic distances and continually updated at every iteration allowing for connectivity changes. These bond orders are incorporated in all valence terms (i.e. energy contributions dependent on connectivity, like valence angle and torsion angle energy) ensuring that energies and forces associated with these terms go to zero upon dissociation. Furthermore, ReaxFF describes non-bonded interactions between all atoms, irrespective of connectivity. Excessive short-range repulsive/attractive non-bonded interactions are circumvented by inclusion of a shielding term in the vdW and coulomb interactions. ReaxFF aims to provide a transferable potential, applicable to a wide range of chemical environments. To ensure its transferability, the following general guidelines were adopted:

- No discontinuities in energy or forces, even during reactions.
- Each element is described by just one force field atom type. The ReaxFF metal oxide oxygen is described by the same parameters as the ReaxFF oxygen in organic molecules. ReaxFF does not have separate *sp* ^2^ and *sp* ^3^ atoms for carbon; the method determines the atoms hybridization from its chemical environment.
- No pre-definition of reactive sites is necessary: given the right temperature and chemical environment reactions will happen automatically^42^.

ReaxFF studies have been reported for a wide range of materials, including hydrocarbons^36^, nitramines^37^, ceramics^40^, (Si/SiO_2_), metals and metal oxides^38^, metal/hydrocarbon interactions^37^, and metal hydrides^40^ demonstrating that ReaxFF has the versatility required to capture the complexity of the mixed metal catalyst system. Meanwhile, the ReaxFF potentials are demonstrated to be about two order of magnitude more expensive than conventional force fields such as CHAARMS and AMBER, but are remarkably faster (several orders of magnitude) than ab initio calculation methods.

In the present work, there are 50 CO_2_ and 50 H_2_ molecules in the simulation system with the dimension of 18 Å × 22 Å × 120 Å and periodic boundary conditions (PBC) were applied in all three dimensions. The gas box involved H_2_ and CO_2_ gas molecules and two pore graphene sheets were located in both sides of the gas mixture to enhance the efficiency of gas permeability of nanoporous membranes. The porous graphene membranes were located in the middle of the simulation box. It should be noted that there is vacuum space besides two sides of the graphene membranes. The molecules of each gases have been randomly positioned within the simulation box between the nanoporous graphene membrane. The length and width of modeled simulation box match to the length of each side of the nanopore graphene membrane (~2 nm). To avoid displacement of the graphene layers due to the pressure exerted by the gas molecules, the position of two carbon atoms in the sheet have been fixed during the simulation procedure. The selected atoms are located as far away as possible from the pores to simulate realistic behavior of atoms in the neighborhood of the rim.

We performed a constant-volume/constant-temperature dynamics (NVT) simulation at 300 K controlled by the Andersen thermostat method, with a fix time step of 0.5 fs. The vdW interactions were applied with a cut off distance of 12 Å. The Ewald sum scheme was employed to describe the electrostatic interactions. Every modeled system was simulated for a 4 *n*s MD simulation, and data were collected at each 0.05 ps. The total energy and the temperature of systems were found to be stable over 20 ps.

## Results and Discussions

### Interaction of Gas Molecules with Graphene Surface

We first calculate the structural properties of H_2_, CO_2_ and graphene systems by molecular mechanics based ReaxFF potential and compared the results with the DFT**-**B3LYP methods. For this aim, a graphene flake consisting of 32 C and 14 H atoms as a suitable model for graphene was considered. Full structural optimization of all molecular systems was performed and the results indicate that there is good agreement between the two calculation results. The structural parameters obtained with considered methods are listed in Table 1a. The calculated structural parameters are also in good agreement with the experimental values^48–50^, (see Table 1a). We have also calculated the charge populations which can play an important role in binding nature of interacting entities. The charge population analysis was performed for CO_2_ molecule with the DFT calculation by Mulliken and NBO approaches and also through the ReaxFF approach with QEq scheme^51^. The calculated charges are listed in Table 1b. Charge analysis reveals that ReaxFF results qualitatively agree well with the reliable NBO values while the obtained various values agree to a reasonably close approximation. It was also found that Mulliken analysis gives a poor result quantitatively against to the NBO analysis.

**Table 1:** Calculated (a) structural geometry parameters with DFT-B3LYP and ReaxFF methods for CO_2_, H_2_ and graphene systems and corresponding experimental values and (b) charge population analyses for a CO_2_ molecule by DFT and ReaxFF approaches.

(a)			
	**C**=**O (Å)**	**H-H (Å)**	**C-C (Å)**
ReaxFF	1.158	0.743	1.423/1.411
DFT-B3LYP	1.160	0.744	1.421/1.416
Exp.	1.162	0.74	1.420/1.415
**(b)**			
	**QEq-ReaxFF**	**NBO-DFT**	**Mulliken-DFT**
O	−0.29	−0.49	−0.19
O	−0.29	−0.49	−0.19
C	0.58	0.98	0.38

**Figure 1:**
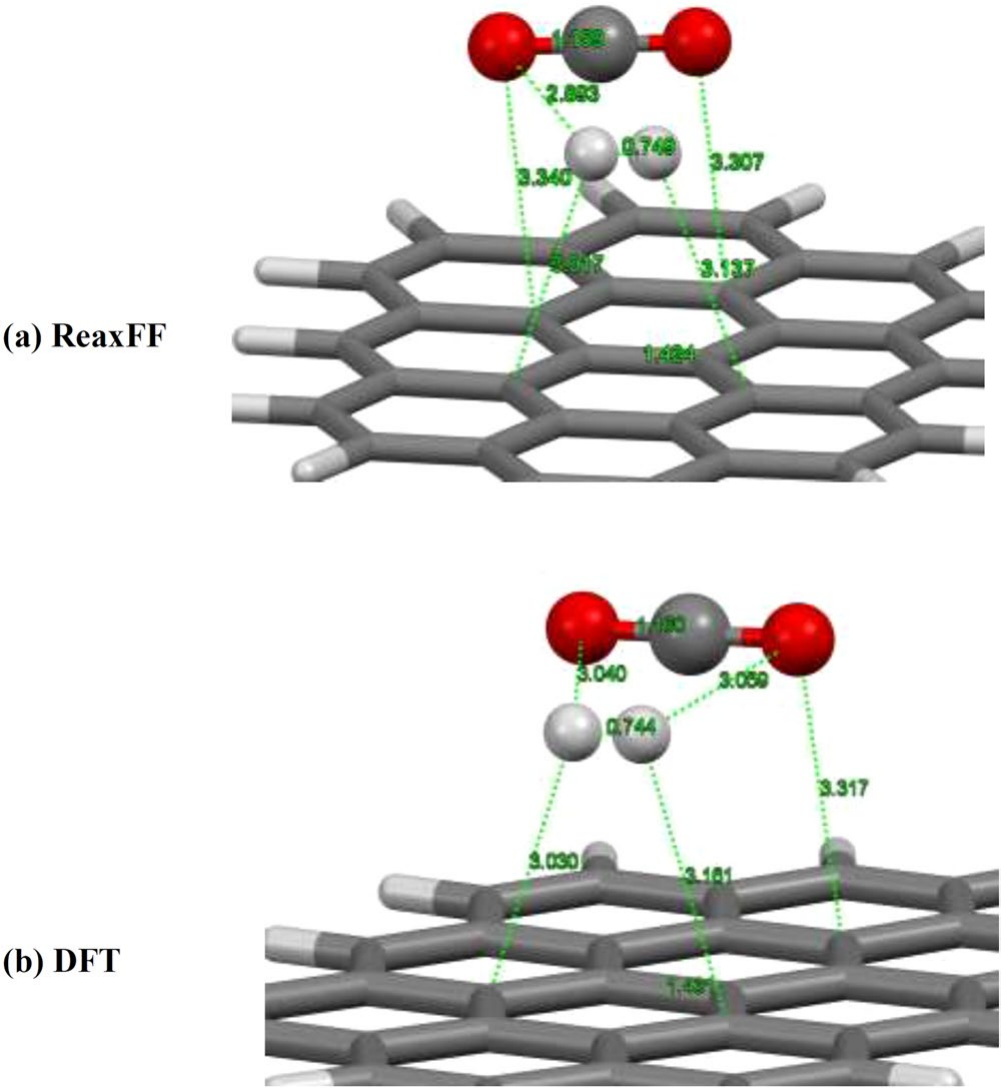
Optimized geometries of CO_2_/H_2_ molecules interacting with graphene surface with (**a**) ReaxFF and (**b**) DFT-B3LYP methods.

We next investigate the interaction properties of single H_2_/CO_2_ molecule with graphene surface by ReaxFF potential and compared with those of the DFT-B3LYP level of theory as a rule of thumb for reasonable reliability. The H_2_ and CO_2_ molecules were placed over the graphene flake surface with their molecular axes parallel to the sheet. The full view of modeled system is represented in Fig. 1. Full structural optimization was carried out for the system under study by two considered calculation methods, i.e., ReaxFF and DFT-B3LYP. After full structural optimization of the whole system with the ReaxFF, we found that both H_2_ and CO_2_ molecules float on the graphene surface with equilibrium distance of about 3 and 3.3 Å, respectively (see Fig. 1(a)). The results obtained by the DFT calculation with B3LYP-D3/TZVP theoretical model demonstrate similar geometrical properties for the interacting entities. Further, the equilibrium distances between H_2_ and CO_2_ molecules are calculated and the results indicate that there is good agreement between the ReaxFF potential and the DFT-B3LYP results. The calculated bonds length values for the adsorbed H_2_ and CO_2_ molecules were determined to be almost the same with both theoretical methods. These reasonable agreements of ReaxFF with DFT-B3LYP method are adequate to describe structural geometries and interaction properties of systems under consideration with the ReaxFF potential.

### MD Simulations for Gas Separation

**Figure 2:**
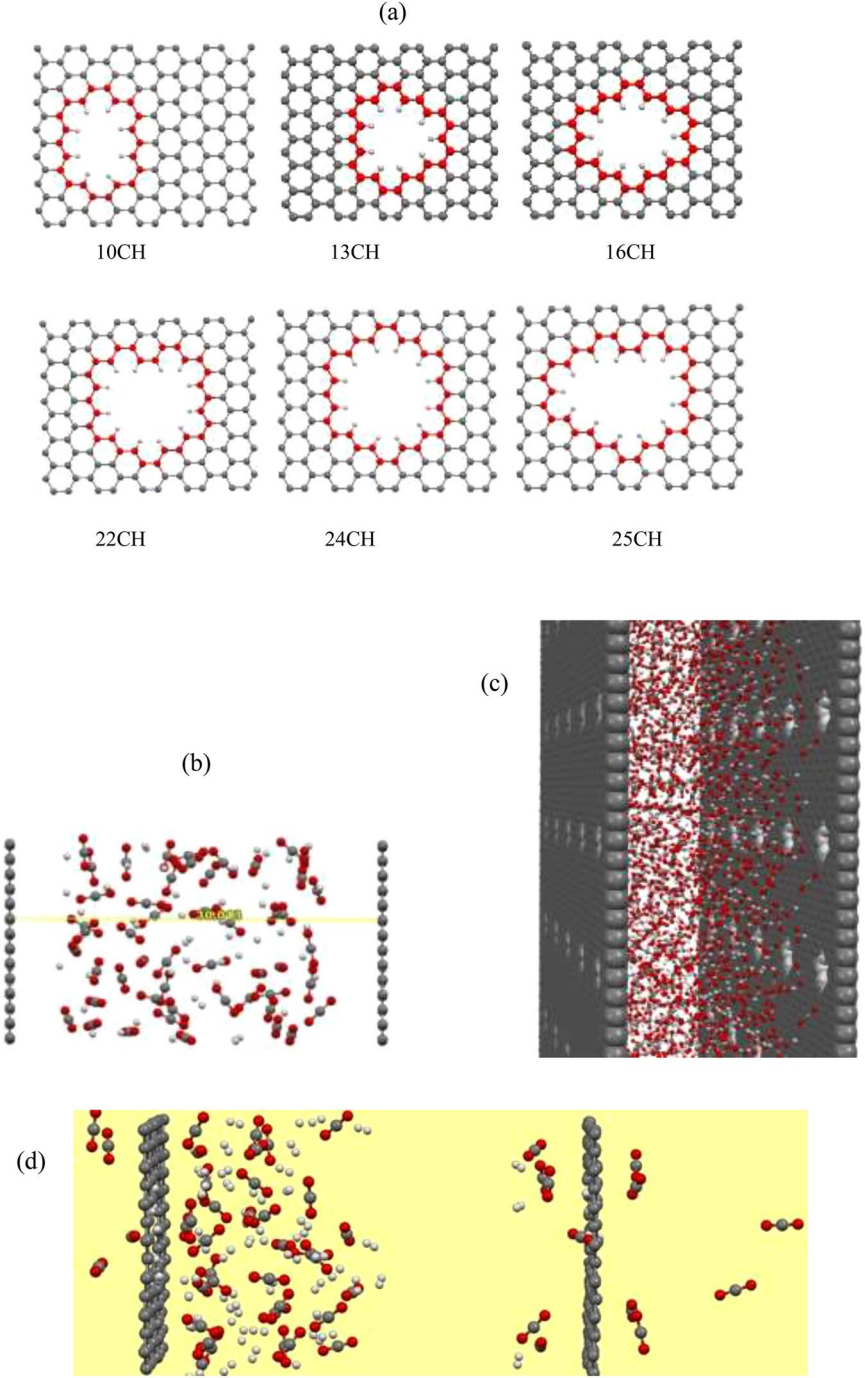
Schematic representation of (**a**) different pore sizes and shapes of graphene membrane, and (**b**) double layers graphene membrane containing H_2_/CO_2_ mixture. (**c**) The supercell model for graphene membrane containing H_2_/CO_2_ system. (**d**) Snapshot of H_2_/CO_2_ mixture between graphene sheets with pore 16CH at 10 ns simulation time.

We then designed some models of double layer porous graphene with different sizes and functional atoms that placed in distance of 30 Å from each other. The schematic representation of the considered porous graphene models is shown in Fig. 2. As it can be seen from the figure, a series of pore sizes were gained by removing (drilling) the carbon atoms from the graphene lattice. The porous graphene models were named according to the number of removed/added C/H atoms, i.e., 10CH (pore-10 with H capped edge), 12CH and so on. To investigate the effect of size and shape of the pore on CO_2_/H_2_ separation, it is important to determine what kind of pore has a good balance between the selectivity and permeability. Permeability is the flux of a specific gas passing through the membrane, and the selectivity (the permeation ratio) is the ratio of the number of permeation events of the two types of gas molecules. It can be inferred that if the permeation ratio equal to one, there is no selectivity and the higher ratio means higher selectivity. Pore size was specified as the mean of the shortest and largest inner distances in the pore and the pore area was calculated according to the area of benzene rings drawn out^24^ (Table 2). The flow is defined to determine the membrane permeability quantitatively as below:

3 $${\rm{F}}=N({\rm{mol}})/S({{\rm{m}}}^{2}).t({\rm{s}})$$

where *N* is the moles of gas molecules that permeate thorough the membrane in both direction, *S* is the area of membrane in total and *t* is the time duration.

**Table 2:** Pore size, pore area, the number and flow of gas molecules passing through the grapheme porous membrane and, the selectivity of considered sub-nanometer pores.

Name	Size (Å)	Area (Å^2^)	H_2_-passage	CO_2_-passage	Selectivity (CO_2_/H_2_)	Flow×103 (mol/m^2^.ns) (F to CO_2_)	Flow×103 (mol/m^2^.ns) (F to H_2_)
**10CH**	3.27	8.76	0	3	∞	7.11	0
**13CH**	3.49	11.17	0	6	∞	11.15	0
**16CH**	3.75	12.89	0	7	∞	11.28	0
**22CH**	4.04	15.47	2	9	4.4	12.08	2.68
**24CH**	4.50	17.19	4	14	3.5	16.91	9.66
**25CH**	4.73	17.86	14	17	1.21	19.76	16.28

We first consider the pore 10CH membrane on CO_2_/H_2_ separation performance (the pore size of 3.27 Å which is close to the kinetics molecular diameter of selected molecules). After 4 *n*s MD simulation the number of passing molecules across the membrane is estimated and listed in Table 2. It can be seen that pore 10CH membrane shows a good selectivity of CO_2_ molecules for CO_2_/H_2_ mixture. We next investigate the influence of pore size on the separation process. Different pore sizes of porous graphene saturated with hydrogen are considered (see Fig. 1) and corresponding MD simulations results are given in Table 2. It was found that H-passive membranes with pore sizes of 3.45 and 3.75 Å, respectively for pores 13CH and 16CH exhibit higher selectivity than the 10CH counterpart. It should be noted that the permeability of 16CH pore is rather larger than 13CH one. Further, our MD simulations demonstrate that pore 22CH with short side of 4.04 Å has lower selectivity than above mentioned pores though its permeability is found to be higher than the smaller pores. We find the lower selectivity and higher permeability with increase in the pore size (24CH and 25CH with pore sizes of respectively 4.50 and 4.73 Å). This is because the pore size is large enough for both CO_2_ and H_2_ molecules to permeate. As a result, the pore 16CH with size of 3.75 Å seem to be the best candidate for CO_2_/H_2_ separation with higher selectivity than the other considered pores. The pore 10CH with 3.27 Å size exhibits rather high selectivity while its less permeability make it undesirable for CO_2_/H_2_ separation. Recently, Tao *et al*. investigated separation of H_2_/N_2_ and H_2_/CO mixtures and showed that all the gases systems reached a balanced state in 3−4 ns^52^. They performed an extended time up to 20 ns and found that a 5 ns simulation time is long enough for such gases systems to obtain some regular results. We have further evaluated extended simulation time for optimal pore membrane, 16CH pore, and found that there is no H_2_ passing through the pore even after 10 ns. Figure 2(d) represents the snapshot of 16CH pore membrane containing CO_2_/H_2_ mixture after 10 ns of simulation time. This superior selectivity might be attributed to the attraction between the positive sites of the rim (H atoms) and O atoms of CO_2_ molecules which causes the CO_2_ to migrate toward the pore while these positive sites repel the H_2_ molecules from the pore edge.

### Mechanism of Gas Molecule Penetrating across 16CH Pore

**Figure 3:**
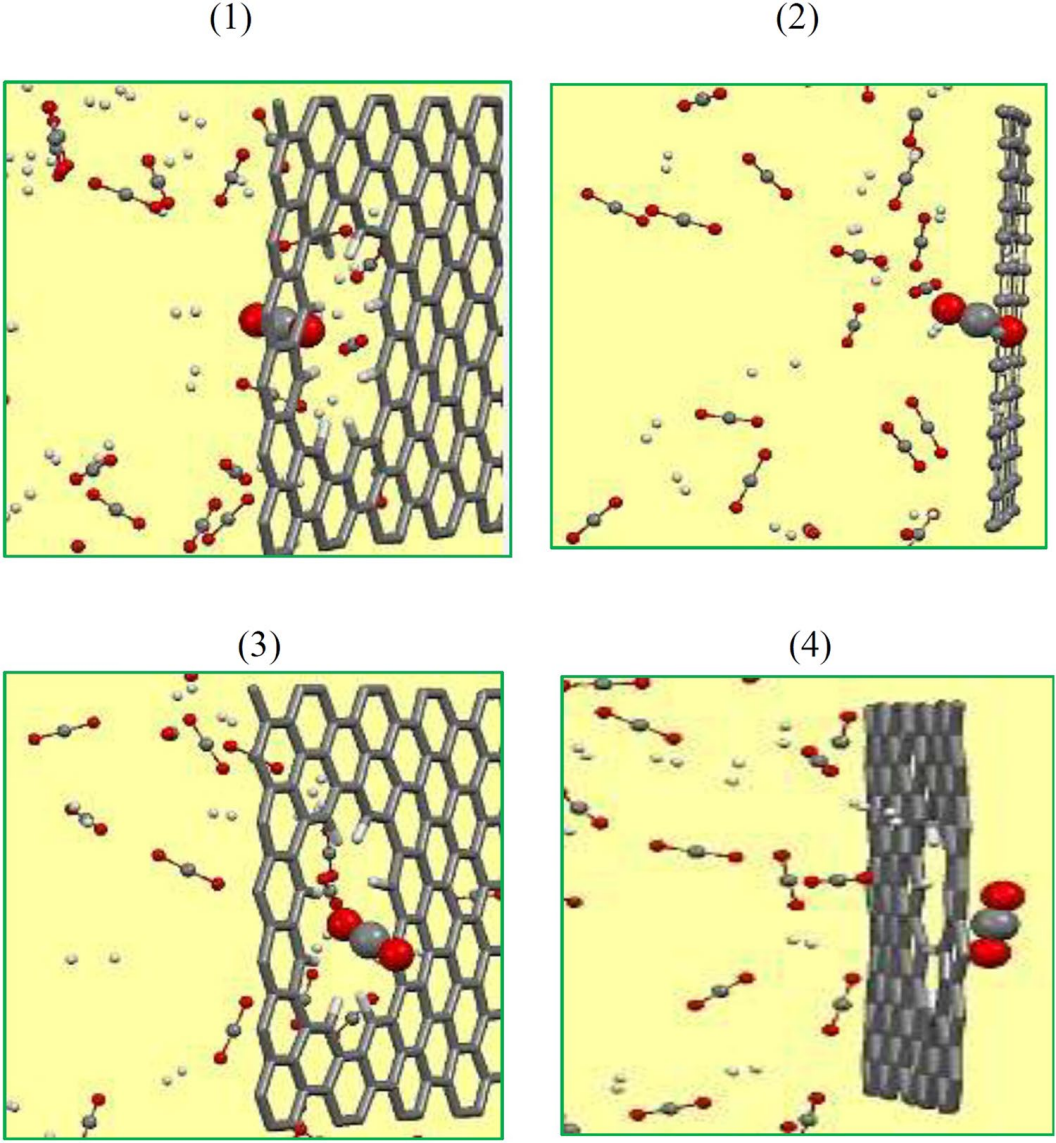
Snapshots of a CO_2_ molecule passing through the pore 16CH.

**Figure 4:**
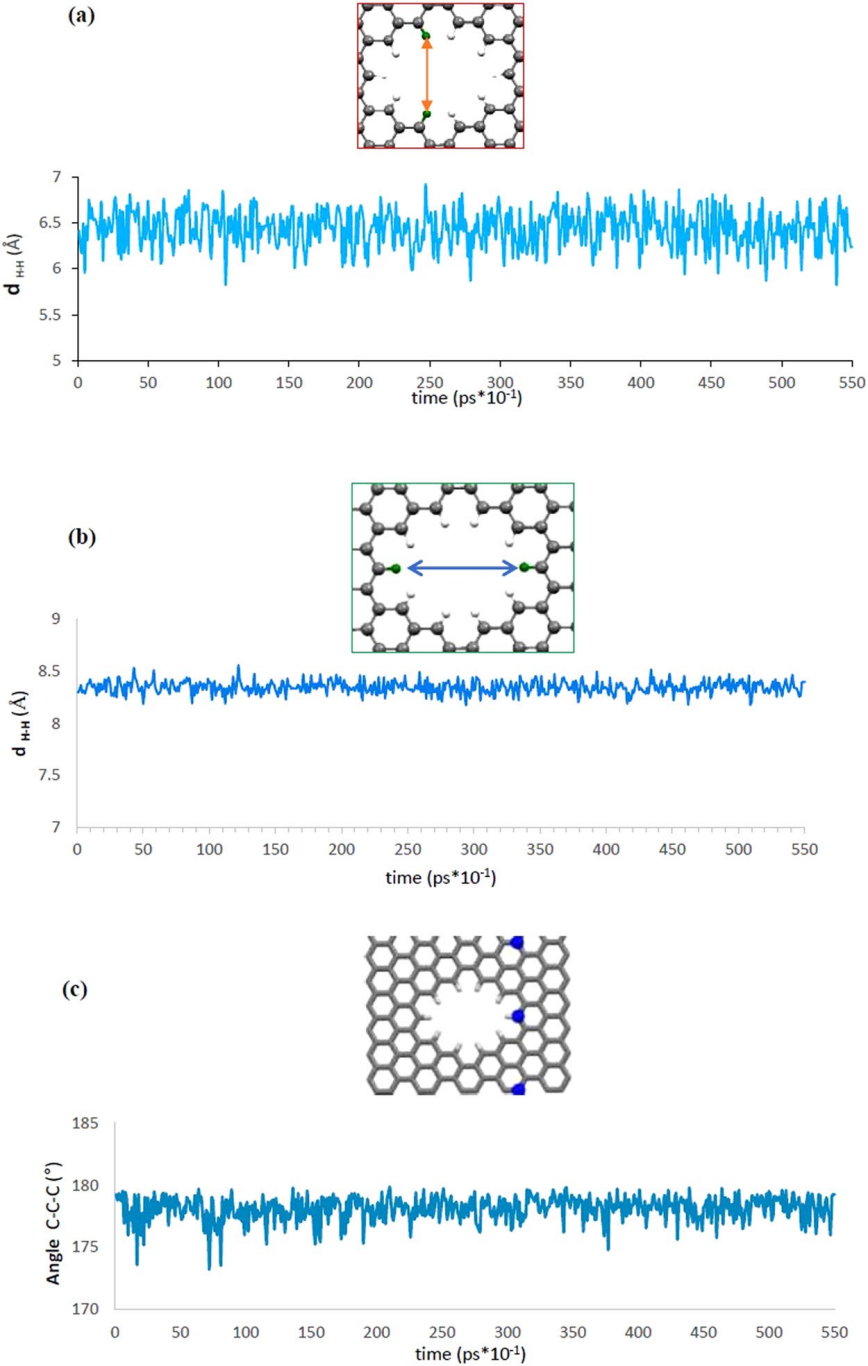
Calculated equilibrium distances between (**a**) horizontal, (**b**) vertical H atoms of the rim and, (**c**) C atoms around the pore during the simulation time 0–55 ps.

We now explore the progress of CO_2_ passing through pore 16CH porous graphene. According to the MD simulation observation, we can divide the permeation process into three steps. At the first, the molecule moves close to the membrane pore. Then it shifts and moves back and forward several times for some picoseconds, and then goes to the other side of the membrane. During the simulation time, both CO_2_ and H_2_ molecule approach the graphene surface. After that one CO_2_ molecule moves back and forth around the pore and finally overcomes the barrier energy and permeates vertically through the pore at 43 ps of simulation time. The snapshot of passing progress of a CO_2_ molecule through the pore 16CH is represented in Fig. 3. After some picoseconds more and more CO_2_ molecules pass through the pore while after 10 ns of simulation time there is still no H_2_ transition which shows really high selectivity of 16 H pore for CO_2_/H_2_ separation. We have also addressed the changes in the pore conformation when the gas molecules passed through the membrane. To this end, the distances between two vertical/horizontal hydrogen atoms as well as the angle between three C atoms around the pore during the simulation time have been calculated (see Fig. 4). Comparing the equilibrium distance between two H atoms shows that the vertical H atoms suffer more fluctuations than that of the horizontal ones. This finding indicate that vertical H atoms (which is attributed to the short side of the pore) can play a key role in the gas penetration through the pore. Furthermore, the graphene sheet deviates from the flatness where surface angle fluctuates around 5° during the simulation times as depicted in Fig. 4(c).

**Figure 5:**
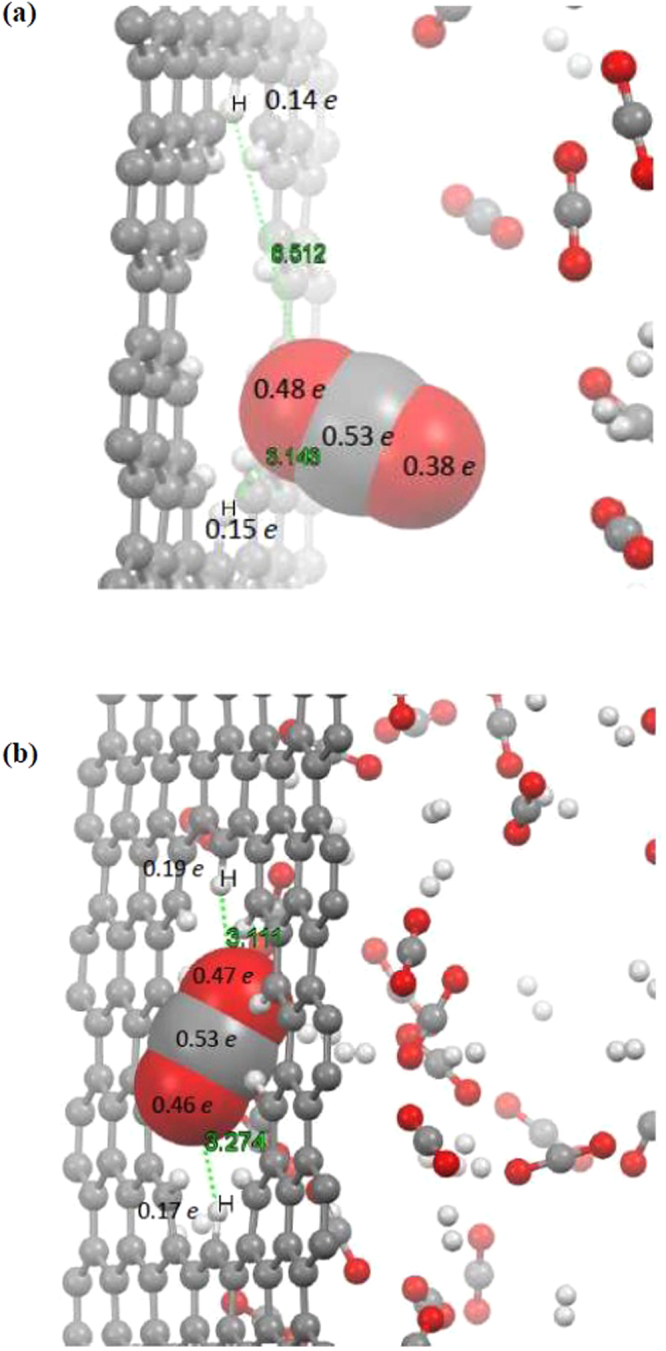
Calculated charge population for passing CO_2_ molecule and hydrogen atoms of the pore rim for 16CH graphene membrane with ReaxFF-QEq scheme.

To gain further insight into the interaction nature of system under consideration we dedicate the charge transfer between the passing molecule through the pore and the adjacent atoms of the rim. Thanks to the ReaxFF potential employed in the present work, it is now possible to evaluate the amount of transferred charges during the penetration process. The QEq scheme was employed for charge analysis between passing CO_2_ molecule and H atoms of the pore at 1 ps (CO_2_ molecule is located far apart the pore) and 10 ps (CO_2_ molecule is trapped in the pore). Figure 5 represents the amount of charge located on the selected atoms. Our reactive potential analysis shows that accommodated charge on the O atoms of CO_2_ was enhanced when it locates between the H atoms of the pore. Indeed, a significant charge has been transferred from the H atoms of the rim to the O atoms of the trapped CO_2_ molecule in the pore during the penetration process.

### Barrier Energy for Gas Molecules Crossing 16CH Pore Membrane

**Figure 6:**
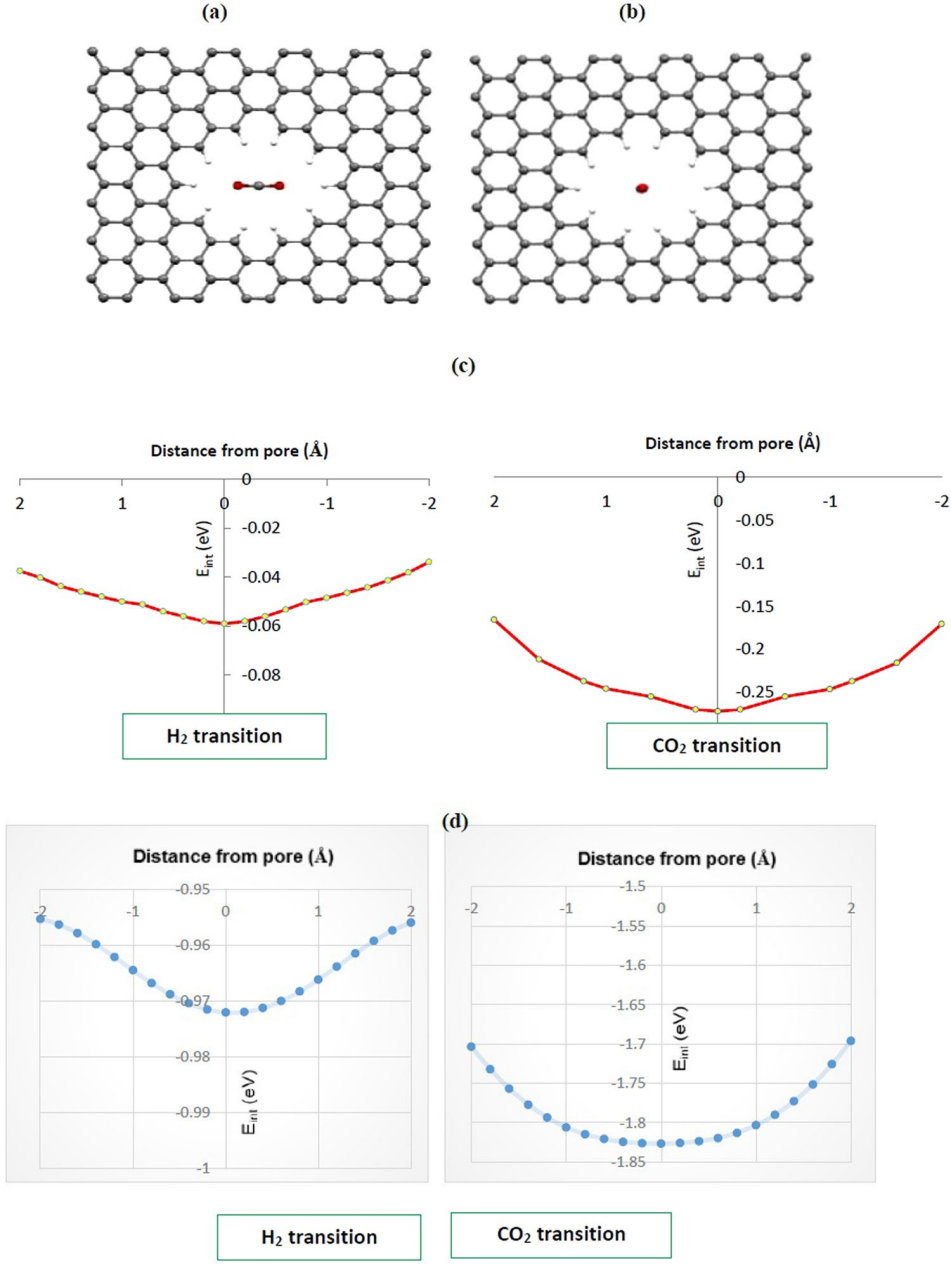
Two interaction configurations for CO_2_ molecule approaching to the center of the pore 16CH with (**a**) molecular axis parallel and (**b**) perpendicular to the plane of the graphene sheet. Calculated interaction energies for H_2_ and CO_2_ molecule molecules passing through the 16CH pore with (**c**) DFT-D and (**d**) ReaxFF methods.

We now investigate the gas separation mechanism by quantum mechanics calculations at the DFT-D level of theory and compare the results with the ReaxFF potential ones. We have considered two configurations for a H_2_/CO_2_ gas molecule passing through the center of the pore; i.e., parallel and perpendicular orientations for molecular axis of approaching molecule toward the center of the pore. We considered 16CH pore as the optimal pore for the penetration mechanism. Figure 6 shows the selected configurations for a CO_2_ molecule approaching the graphene pore. The calculated interaction energies are given in Table 3. Our *first-principles* calculation results show that CO_2_ molecule prefers to pass through the pore 16CH with its molecular axis perpendicular to the graphene surface. The calculated *E*_barr_ for CO_2_ molecule passing through the pore with perpendicular orientation was 0.17 eV while in the parallel one was about 0.19 eV (see Table 3). The results obtained from ReaxFF are qualitatively in agreement with the DFT results (0.3 and 0.8 eV for perpendicular and parallel orientations, respectively). This finding based on the DFT calculation confirms our reactive MD simulation results of Fig. 3. Indeed, the perpendicular orientation is most possible for CO_2_ molecule to penetrate across the membrane pore. For comparison the calculated interaction energies versus the interaction distances by using DFT-D and ReaxFF methods for CO_2_ and H_2_ molecules passing through the 16CH pore in perpendicular orientation were demonstrated in Fig. 6. As it can be seen from the curves for both molecules the interaction is attractive and as the distance from the pore (interaction distance) increases, the interaction energy and attractive force decreases. In addition, the calculated interaction energies with both DFT-D and ReaxFF methods follow the same trends so that the gas molecules reach a shallow attractive well at the center of the rim.

**Table 3:** Calculated barrier energies, *E*_bar,_ with both DFT-D and ReaxFF methods for H_2_ and CO_2_ molecules passing through the 16CH pore membrane.

Method	Gas	16CH-Perp	16CH-Par
ReaxFF	CO_2_	0.30	0.83
	H_2_	0.02	0.02
DFT	CO_2_	0.17	0.19
	H_2_	0.08	0.04

The obtained barrier energy for the H_2_ molecule passing through the 16CH pore was found to be smaller than the CO_2_ molecule which indicates easier penetration of hydrogen molecule across the membrane (see Table 3). However, as we can find from the MD simulation results the CO_2_ molecules permeate through the pore while H_2_ molecules were rejected. This discrepancy can be explained by this fact that the interaction energy play an important role in the penetration of gases molecules through the pore. As it was found from the interaction energies, the calculated *E*_int_ for the CO_2_ molecule is higher than the H_2_ one which cause a stronger interaction between the pore rim and the CO_2_ molecule than the H_2_ molecule. This causes CO_2_ molecule to be highly attracted to the pore rim and then inserted pressure from other gases push the trapped molecule to easily penetrate through the pore. Meanwhile, CO_2_ has a significant quadrapole moment enhancing dispersive attraction with hydrogen modified graphene which increase the attractive interactions. These confirm the MD simulation results where only CO_2_ molecules can pass through the pore in 16CH membrane. Our findings completely are in agreement with the reactive MD results which indicate a high selectivity of pore 16CH for CO_2_/H_2_ separation.

## Conclusion

In summary, by drilling the graphene lattice and functionalizing the pore rim as well as considering double layers pore strategy, we significantly enhanced the selectivity and permeability of nano-porous membranes for CO_2_/H_2_ separation. We have investigated the performance of porous graphene with various pore size for separating CO_2_/H_2_ mixture by using the reactive MD simulation. Our MD simulation results showed that H-passive membranes with pore size (short side) of 3.75 Å (16CH pore) performs high selectivity and desirable permeability for CO_2_/H_2_ separation while smaller and larger pores demonstrated less permeability and selectivity, respectively. The 16CH pore was found to be optimal porous membrane among the considered pores and then 13CH pore (short side of about 3.5 Å) with rather the same selectivity but less permeability. The reactive MD simulations demonstrated that CO_2_ molecules were trapped into the pore and have a long delay time during the penetration due to the attraction between O atoms of the CO_2_ and H atoms of the pore. Charge analysis by ReaxFF based QEq scheme which is validated by DFT-NBO level of theory indicated that significant charges were transferred from the H atoms of the pore to the O atoms in the trapped CO_2_ molecule during the penetration process. This reveals rather strong interaction between passing CO_2_ molecules and pore membrane.

The mechanism of gas separation was investigated within DFT-D calculations. Using the *first-principles* calculations it was found that CO_2_ prefer to pass through the 16CH pore with its molecular axis perpendicular to the graphene pore. However, the difference between calculated barrier energies of CO_2_ and H_2_ is high (less barrier energy for H_2_ molecule) while the results showed high permeability to CO_2_ molecules and no H_2_ molecules allowed to permit. This observation attributed to higher interaction energies and so higher attraction between CO_2_ molecules and H atoms of the pore which cause the CO_2_ molecules to be adsorbed to the pore rim. Finally, reinforce pressure of other gases molecules as driving force push these trapped molecules to easily penetrate through the pore.

Therefore, modified pores with 3.75 Å of size (short side) accompanied with double layer strategy provide a promising approach for constructing new materials with innovative separation properties. Researches on CO_2_ separation have numerous applications such as industrial applications, CCS (CO_2_ capture and storage), and environment protection. Future works are dedicated on developing novel hetero atoms nanosheets such as h-BN and ZnO mono-layers as well as functionalized graphene and applying them in gas separation.
